# Nutritional Status of Very Elderly Outpatients with Heart Failure and Its Influence on Prognosis

**DOI:** 10.3390/nu16244401

**Published:** 2024-12-21

**Authors:** Sonia González-Sosa, Pablo Santana-Vega, Alba Rodríguez-Quintana, Jose A. Rodríguez-González, José M. García-Vallejo, Alicia Puente-Fernández, Alicia Conde-Martel

**Affiliations:** 1 Internal Medicine Department, Hospital Universitario de Gran Canaria Dr. Negrín, 35010 Las Palmas de Gran Canaria, Spain; sonia.gonzalez112@alu.ulpgc.es (S.G.-S.); jberto6@gmail.com (J.A.R.-G.); jogava90@gmail.com (J.M.G.-V.); alicia.puente@ulpgc.es (A.P.-F.); 2 Health Sciences Faculty, Universidad de Las Palmas de Gran Canaria, 35016 Las Palmas de Gran Canaria, Spain; pablosv2000@gmail.com (P.S.-V.); infbalbarodriguez@gmail.com (A.R.-Q.)

**Keywords:** heart failure, elderly patients, nutritional status, malnutrition, survival

## Abstract

Background/Objectives: Malnutrition has been associated with increased morbidity and mortality in elderly patients diagnosed with heart failure (HF). However, nutritional problems are underdiagnosed in these patients. This study aimed to analyse malnutrition prevalence in elderly HF patients and its impact on survival. Methods: We conducted a retrospective observational study including patients aged ≥85 years diagnosed with HF followed up by a specific HF unit between 2015 and 2023. All patients underwent a nutritional assessment at the start of follow-up. Demographic characteristics, comorbidities, functional, cognitive and frailty status, heart disease characteristics and laboratory data, as well as admissions, emergency department visits and survival, were collected. The sample was categorised according to nutritional status into normonutrition and impaired nutritional status, and differences were evaluated. Results: Of a total of 413 patients, 52.8% were female, and the mean age was 88.4 ± 2.9 years. A total of 25.4% were at risk of malnutrition and 2.2% malnourished. Dementia [OR = 3.99, 95%CI (2.32–6.86); *p* < 0.001], hip fracture [OR = 3.54, 95%CI (1.75–7.16); *p* < 0.001)], worse Barthel index score [OR = 5.44, 95%CI (3.15–9.38); *p* < 0.001), worse Pfeiffer test [OR = 5.45; 95%CI (3.29–9.04); *p* < 0.001), worse Frail index [OR = 6.19; 95%CI (2.45–15.61); *p* < 0.001] and higher Charlson index [OR = 1.95; 95%CI (1.21–3.15); *p* = 0.006] were associated with worse nutritional status. In addition, patients with poor nutritional status lived 16.69 months less (*p* < 0.001) than normonutrited patients. Conclusions: At least one in four elderly patients with HF under outpatient follow-up has an impaired nutritional status. This is associated with hip fracture and greater functional and cognitive decline. Patients who are malnourished or at risk of malnutrition survive less than those who are not malnourished.

## 1. Introduction

Heart failure (HF) is a clinical syndrome resulting from a structural or functional alteration that produces inadequate cardiac output at rest or during exercise. The incidence was estimated at 2.78 cases per 1000 person-years, with a slight increase in recent years in relation to the ageing of the population and the increase in cardiovascular risk factors [1]. Specifically, in the population over 80 years of age, a prevalence of 9% has been described [1]. It is, therefore, a serious health problem in Spain, not only because of its high incidence and prevalence but also because it has a high mortality rate and causes high costs to the health system [2].

Malnutrition is one of the many factors that interact to shape outcomes for patients with heart failure [3]. In fact, nutritional risk and malnutrition are strongly associated with a significant decline in quality of life, higher morbidity and mortality, and longer hospital stays among elderly patients [3,4,5]. Many patients are malnourished prior to hospital admission, a condition that frequently worsens during their stay due to underlying pathological conditions and the associated loss of autonomy [5].

The prevalence of undernutrition in the elderly varies according to multiple factors. For example, elderly people living in the community have a risk of undernutrition of 7.8%, rising to 28.4% in nursing homes and reaching 56% in long-stay facilities [6].

The prevalence of malnutrition or the risk of malnutrition is even higher in patients with HF, particularly in patients with advanced and acutely decompensated HF [7]. Although the precise biological mechanisms behind malnutrition in HF patients remain unclear, several contributing factors have been identified. These include elevated inflammatory cytokines, a hyperadrenergic state with heightened metabolic requirements, and physical inactivity. Together, these factors drive processes such as increased catabolism, poor appetite, reduced anabolic activity, peripheral vasoconstriction, malabsorption due to intestinal oedema and muscle mass loss [8,9,10]. Malnutrition has been associated with poor prognosis and increased mortality in this group of patients [7,8].

All of the above makes a detailed nutritional assessment very necessary in this population group. However, in the health sector, there is little awareness of the problems of nutrition in the elderly [11]. As a consequence, there is under-diagnosis, which in many cases does not allow an adequate approach to be taken [11]. Lack of diagnosis means lack of treatment, which could lead to increased mortality and hospital stays, as well as increased healthcare costs [3].

Therefore, this study aims to evaluate the nutritional status of elderly HF patients who are enrolled in an outpatient follow-up programme. Additionally, the study seeks to identify characteristics of patients who meet malnutrition criteria and to examine the association between malnutrition and various clinical and sociodemographic factors. Finally, the study aims to determine whether there is a relationship between undernutrition and increased mortality rates.

With the results obtained, we aim to provide valuable information for improving or reinforcing comprehensive care and management for these complex patients.

## 2. Materials and Methods

### 2.1. Design, Study Population and Follow-Up

We conducted a retrospective observational study utilising data retrieved from electronic health records. The research methodologies adhered to the guidelines outlined in the STrengthening the Reporting of OBservational studies in Epidemiology (STROBE) [12].

We included patients diagnosed with heart failure who were being followed up between 2015 and 2023 by the Unit for the Integrated Management of Patients with Heart Failure (UMIPIC) of the Internal Medicine Department of the University Hospital of Gran Canaria Dr. Negrín. Patients were reviewed through electronic data until the date of exitus or December 2023, inclusive.

### 2.2. Inclusion Criteria

Patients who were included in the study had to be 85 years of age or older at the time of their first outpatient evaluation and to have been diagnosed with HF according to the criteria of the European HF guidelines [13].

### 2.3. Exclusion Criteria

Patients who did not undergo a nutritional assessment at the start of follow-up were excluded from the study. The main reason for not having a nutritional assessment was lack of time at the clinic.

### 2.4. Data Collection

Data were obtained from digital medical records and subsequently compiled and analysed from a secure, anonymised electronic database.

Sociodemographic characteristics were collected, including sex, age, place of residence and cohabitants, as well as the patient’s comorbidities and toxic habits. The short-form questionnaire—Mini Nutritional Assessment (MNA-SF) [14] was used to record the nutritional status of the patients. This validated assessment questionnaire consists of six questions with several answers scored from 0 to 3 points and allows the patient to be classified in normal nutritional status if they have a score between 12 and 14 points, at risk of malnutrition if they have a score of 8–11 points and malnourished with a score equal to or less than 7 points [14]. In addition, functional status was assessed using the Barthel index [15], cognitive status using the Pfeiffer test [16] and frailty using the Frail index [17].

In relation to heart failure, we recorded whether it was a debut or known HF, the aetiology (ischaemic, hypertensive, valvular, infiltrative, etc.), the left ventricular ejection fraction (LVEF) assessed by transthoracic echocardiography, the functional grade according to the New York Heart Association (NYHA). In addition, analytical data were collected, including haemoglobin, renal function, ions and NT-proBNP, as well as treatment at 3-month follow-up consultation. In addition, emergency department (ED) visits and admissions in the year prior to follow-up and subsequent evolution after follow-up in consultation were recorded, including ED visits, hospital admissions, referrals to the Palliative Care Unit, and death until December 2023.

### 2.5. Statistical Analysis

Statistical analysis was performed with SPSS (Statistical Package for the Social Sciences, IBM Corp., Version 29.0, Armonk, NY, USA). Categorical variables were expressed as frequencies and percentages, and quantitative variables as mean and standard deviation (SD) or as median and interquartile range, depending on whether or not the distribution was normal, which was assessed using the Kolmogorov–Smirnov test.

Subsequently, a univariate analysis was performed. Differences between patients were analysed according to their nutritional status. The Chi-squared test or Fisher’s exact test was used to assess the relationship between categorical variables, and, for the relationship with quantitative variables, the Student’s *t*-test or Mann–Whitney U test was used depending on whether or not the variables followed a normal distribution.

Survival was estimated using the Kaplan–Meier method, and the log-rank test was used to compare patient survival according to nutritional status.

To assess whether undernutrition or risk of undernutrition is independently associated with mortality, a multivariate analysis was conducted using a Cox regression model. A value of *p* < 0.05 was considered statistically significant.

### 2.6. Ethical Aspects

The study was approved by the Clinical Research Ethics Committee of the Hospital Universitario de Gran Canaria Dr. Negrín.

This study was conducted according to the guidelines laid down in the Declaration of Helsinki. Personal data were processed in accordance with the provisions of Spanish Organic Law 3/2018, of 5 December, on the Protection of Personal Data and Guarantee of Digital Rights. The information was anonymised in the database to maintain the confidentiality of the data evaluated.

## 3. Results

### 3.1. Descriptive Analysis of the Sample

Of a total of 445 patients potentially eligible for the study, 30 patients were excluded because they did not undergo MNA at the start of follow-up and 2 patients were excluded due to lack of follow-up in the outpatient clinic. Finally, a total of 413 patients were included, 195 (47.2%) were male, and 218 (52.8%) were female, with a mean age of 88.4 years (SD: 2.98), range: 85 to 101 years; median of 88 (interquartile range [IQR]: 86–90). Regarding the place of residence, 75 (18.2%) lived alone, 107 (25.9%) lived with their spouse, and 8 (1.9%) lived in a residence.

The most frequent comorbidities were high blood pressure (398 patients; 96.4%), atrial fibrillation (293; 70.9%) and chronic kidney disease (247; 59.8%). Comorbidity assessed by the Charlson index was higher than 4 points in 24% of the patients (Table 1).

In terms of functionality, 16.7% of the patients were functionally impaired according to the Barthel index. On the other hand, 22.3% were cognitively impaired according to the Pfeiffer test, and 17.8% could be considered frail, according to Frail (Table 1).

**Figure 1:**
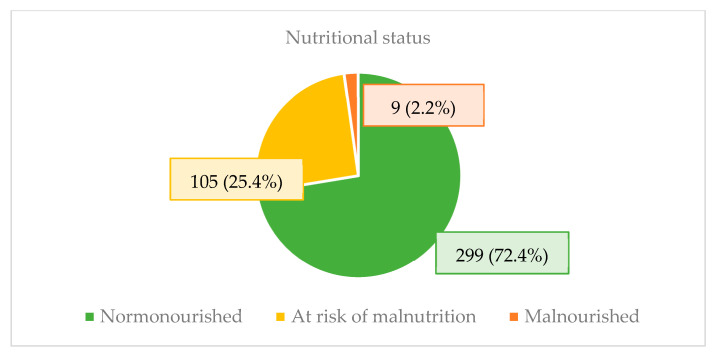
Nutritional status of elderly heart failure patients in outpatient follow-up.

Regarding nutritional status, 72.4% of patients were normonourished, and 114 patients (27.6%) were at risk of malnutrition or malnourished (Table 1; Figure 1).

As shown in Table 2, most patients (308; 75.8%) had heart failure with preserved ejection fraction, and 25.9% of patients were referred at the time of HF debut. The most frequent HF aetiology was hypertensive heart disease (312 patients; 77.0%), and mitral regurgitation was the most frequently diagnosed valve disease (215 patients; 57.0%).

The most frequently used pharmacological treatment at three months of follow-up in consultations was loop diuretics (89.2%) and beta-blockers (63.5%), as shown in Table 2, the drugs collected and their frequency of use.

In relation to hospital admissions, 53.5% were admitted in the year prior to follow-up compared to 10.2% in the year after. Patients were followed for a mean of 27.3 months (SD: 21.7). During follow-up, exitus occurred in 256 (62.0%) of the patients.

### 3.2. Relationship Between Different Variables and Nutritional Status

As shown in Table 3, no significant differences were observed between patients at risk of undernutrition or undernourishment compared to patients with normal nutritional status in terms of age or sex. The percentage of patients living alone was significantly higher among normonutrition patients (OR: 0.51; 95%CI: 0.27–0.96).

There were no differences in the prevalence of the most common comorbidities, such as hypertension, diabetes, or dyslipidaemia. However, dementia (OR: 3.99; 95%CI: 2.32–6.86) and hip fracture (OR: 3.54; 95%CI: 1.75–7.16) were associated with undernutrition or risk of undernutrition. On the other hand, obese patients were associated with less malnutrition (OR: 0.23; 95%CI: 0.13–0.41). However, patients with malnutrition or at risk of malnutrition had higher overall comorbidity as assessed by the Charlson index (Charlson > 4 points: OR: 1.95; 95%CI: 1.21–3.15).

In addition, malnutrition or risk of malnutrition was associated with worse functional status (Barthel ≤ 60: OR: 5.44; 95%CI: 3.15–9.38), worse cognitive status (Pfeiffer ≥ 3 errors: OR: 5.45; 95%CI: 3.29–9.04) and higher frailty (Frail ≥ 3 points: OR: 6.19; 95%CI: 2.45–15.62).

In analytical tests, patients at risk of malnutrition or malnourishment had significantly higher urea levels, lower total protein and higher NT-proBNP.

Table 4 shows that there were no differences in ejection fraction or functional class between the two groups. Regarding HF aetiology, valvular aetiology was significantly more frequent in malnourished patients (OR: 2.05; 95%CI: 1.32–3.19).

There were no differences in terms of admission either before or after initiation of follow-up according to nutritional status.

### 3.3. Survival Analysis

**Figure 2:**
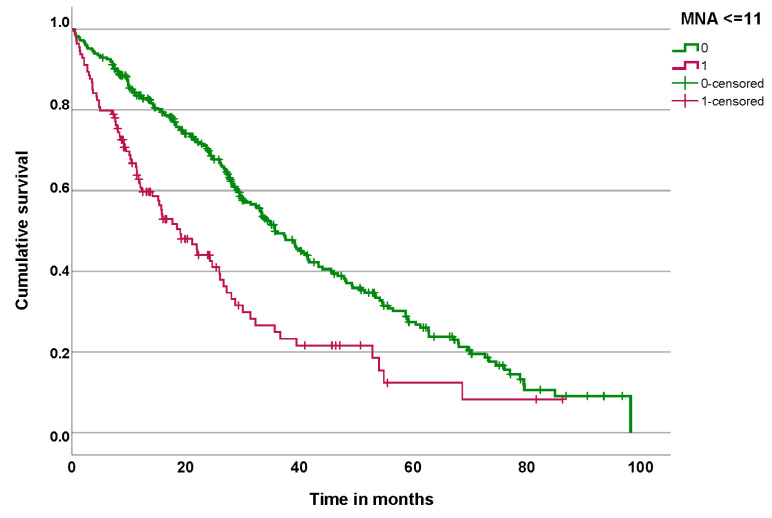
Survival curve of patients at risk of malnutrition or malnourished (red) and normonnourished patients (green).

Patients who were malnourished or at risk of malnutrition had significantly lower survival (*p* < 0.001) than patients with normal nutritional status (Figure 2).

The difference between the median survival of patients who are normonourished and those who are malnourished or at risk of malnutrition is 16.69 months (Table 5).

### 3.4. Multivariate Analysis (Table 6)

Multivariate analysis using the Cox regression model to assess whether nutritional status is an independent predictor of survival, adjusted for sex and age, showed that there was a significant association between worse nutritional status and poorer survival (HR: 1.78; 95%CI 1.31–2.41), with worse functional status also being independently associated with survival using the Barthel index (HR:0.99; 95%CI 0.986–0.999) and greater comorbidity assessed by the Charlson index (HR:1.095; 95%CI 1.03–1.17).

**Table 6:** Factors associated with survival by Cox regression model.

	β	*p*	HR * (CI * 95%)
Age	0.016	0.489	1. 016 (0.971–1.064)
Sex	−0.175	0.206	0.840 (0.641–1.101)
Pfeiffer	0.033	0.370	1.033 (0.962–1.110)
Barthel	−0.007	0.027	0.993 (0.986–0.999)
Charlson	0.090	0.004	1.096 (1.011–1.189)
MNA * ≤ 11 points	0.576	<0.001	1.780 (1.312–2.413)

* HR: hazard ratio; CI: confidence interval; MNA: Mini Nutritional Assessment.

## 4. Discussion

In this series, one in four patients (25.4%) was at risk of malnutrition, and 2.2% of patients were malnourished. The prevalence of malnourished elderly or at risk of malnutrition varies widely among different studies. Articles are found with figures quite similar to those in our series [18,19,20,21]. In fact, a study also conducted on outpatients with HF using the MNA-SF as a screening tool showed a prevalence of altered nutritional status (malnutrition or at risk) of 18.6%, slightly lower than in our series [22]. On the other hand, a meta-analysis [8] evaluated the prevalence of malnutrition in HF patients from 20 articles (7520 patients) with a prevalence ranging from 37% to 56%, the total prevalence being estimated at 46%, higher than that of our study. Additionally, a recent study conducted specifically in HF patients with severe HF showed 35.1% of patients with evidence of malnutrition [23]. Other studies [24,25] show intra-study variations in the percentage of patients with moderate to severe malnutrition depending on the nutritional scoring system used; one study in HF patients ranged from 9.1% to 50% [24]. In a systematic review including 17 studies, the prevalence of undernutrition ranged from 16 to 90%; prevalence differed depending on the test used, the setting (inpatient or outpatient), the severity of HF and comorbidities [5]. However, another study reports lower figures (8.3%) for nutritional status impairment compared to our estimates [26]. These differences could be due to the younger age of inclusion of the patients in the aforementioned study (over 65 years of age) and to the lower comorbidity of the patients, whereas all patients in our series were diagnosed with HF and had multiple associated comorbidities.

Sex was not related to worse nutritional status in our study. However, most published articles do note a higher risk of malnutrition in women [21,27].

Statistically significantly, elderly people living alone were found to have better nutritional status. However, there was no significant association between the risk of undernutrition and undernutrition and whether the elderly lived with a spouse or in a nursing home. Most of the studies reviewed do find a significant association between poorer nutritional status and social isolation, living in a nursing home or requiring home help [6,27,28].

This series of patients had high comorbidity, the most frequent being hypertension (96.4%). This figure is much higher than in other publications, where it is also one of the most frequent comorbidities. This could be mainly due to the age of the patients and probably to the fact that our entire population has heart failure.

In the univariate analysis, an association between dementia and altered nutritional status was observed. There are numerous publications in the scientific literature describing this association [6,29]. Furthermore, poorer mental status, as assessed by the Pfeiffer test, was also associated with poorer nutritional status.

A significant association was observed between poor nutritional status and having suffered a hip fracture. A higher prevalence of malnutrition risk has been demonstrated in elderly patients with hip fractures [30]. In addition, older people with hip fractures and malnutrition have been observed to have a worse functional recovery [31]. Therefore, nutritional intervention in these patients can be very useful to prevent the onset of malnutrition and ensure proper recovery.

Those patients with greater dependency to perform basic activities of daily living, as assessed by the Barthel index, presented worse nutritional status. This association is described in the literature and even with the ability to perform instrumental activities of daily living, assessed by the Lawton and Brody scale not included in our study [6,20,21,32].

Higher comorbidity, as assessed by the Charlson index, was associated with poor nutritional status. This association is evident in other publications [33].

The frail elderly, as assessed by the Frail index, had a higher risk of malnutrition, as also mentioned in other published articles [34].

There was no significant association between previous heart failure admissions or the subsequent year’s risk of malnutrition or undernutrition. However, there are other articles linking malnutrition with increased risk during hospital stays of immune system depression, wound healing problems, muscle atrophy, longer hospital stays, higher treatment costs and higher mortality [35,36]. Joaquín et al. [22] described twice as many crude hospital admissions among HF patients with altered nutritional status compared to those with normal nutritional status, although this relationship was not found to be significant in the multivariate analysis.

In terms of mortality, patients with impaired nutritional status lived 16.7 months less than those with normal nutritional status. Malnutrition status using the Mini Nutritional Assessment score in our study was shown to be an independent predictor of mortality in these patients, as has been described in other studies [37]. Notably, in the multivariate analysis, adjusting for sex, age, comorbidity, cognitive status and functional status, being malnourished or at risk of malnutrition was shown to be an independent predictor of increased mortality. Several studies have demonstrated the lower survival of patients with poorer nutritional status, specifically in patients with heart failure [7,23,25]. In a recent study of patients with heart failure, the estimated rates of all-cause and cardiovascular mortality were significantly higher in patients with malnutrition [23]. A meta-analysis involving more than 10,000 patients with chronic heart failure showed that all-cause mortality was at least twice as high in malnourished patients as in well-nourished patients [8].

This strong link between nutritional status and survival highlights the importance of considering other prognostic factors, such as the clinical hemodynamic phenotype, which can further refine risk stratification in heart failure patients. Clinical guidelines suggest that patients can be clinically classified based on a bedside physical examination, assessing the presence of congestion (classified as “wet” if present or “dry” if absent) and peripheral hypoperfusion (“cold” if present or “warm” if absent). This approach results in four distinct groups: warm and wet (well-perfused with congestion), the most common presentation; cold and wet (hypoperfused with congestion); cold and dry (hypoperfused without congestion); and warm and dry (compensated, well-perfused without congestion). This classification system is valuable for guiding early-phase treatment decisions and offers important prognostic insights [38]. Although the “cold + dry” phenotype is infrequent in our sample, it is worth highlighting the important association between this phenotype and both malnutrition and poor outcomes, as recently reported in very elderly patients with heart failure [39,40].

In general terms and based on the benefits of good nutritional status, this paper aims to emphasise the importance of early nutritional assessment and intervention in nutritional status to improve the management of very elderly patients with heart failure.

The limitations of the study include those inherent to a retrospective observational study and, therefore, the limitation to collecting and verifying all data in all patients and the impossibility of establishing causality. In addition, an initial screening for malnutrition was performed with the abbreviated version of the MNA, which is a valid and reliable method to determine the patient at risk of malnutrition, but a subsequent nutritional assessment and an irrefutable diagnosis of malnutrition is recommended, which was not performed in our study [41]. However, the MNA-SF has been associated with mortality and is an easily usable tool in daily clinical practice [14,22]. As a further limitation, nutritional assessments were conducted only at baseline, so potential changes in nutritional status during follow-up were not accounted for.

However, given the large sample size and the long follow-up of the patients, it is possible to establish valid hypotheses from this study. It would be advisable to carry out and evaluate therapeutic interventions in the nutritional field in order to corroborate our data.

Building on these considerations, this study highlights the critical role of nutritional assessment in the management of patients with heart failure, especially among elderly patients. Given the high prevalence of malnutrition or nutritional risk within this cohort, our findings emphasise the need for routine screening and early intervention to prevent adverse outcomes associated with poor nutritional status, such as functional decline, cognitive impairment and reduced survival rates. In clinical practice, incorporating nutritional assessment tools such as the Mini Nutritional Assessment (MNA) into routine care can help identify at-risk patients and guide therapeutic interventions aimed at improving both their nutritional status and overall prognosis. This study contributes to the growing body of evidence linking malnutrition to poor outcomes in heart failure and underscores the importance of addressing nutritional status alongside other clinical factors to refine risk stratification and optimise patient care.

## 5. Conclusions

At least one in four heart failure patients on outpatient follow-up in a dedicated Heart Failure Unit is either at risk of malnutrition or already malnourished. Impaired nutritional status in these patients is associated with increased dependency on basic activities of daily living, cognitive impairment, hip fracture and valvular aetiology of heart failure. Furthermore, patients who are malnourished or at risk of malnutrition demonstrate lower survival rates compared to those with an adequate nutritional status. These findings highlight the importance of systematically assessing the nutritional status of heart failure patients to detect the risk or presence of malnutrition and implement therapeutic interventions. Such interventions could improve both the nutritional status and the overall prognosis of these patients.

## Figures and Tables

**Table 1:** Overview of assessment scales for comorbidity (Charlson index), functional (Barthel index), mental (Pfeiffer test), frailty (FRAIL index) and nutritional status (MNA).

Assessment Scales				
Charlson index (N = 413)	>4 points [N (%)]	99 (24.0)		
	means ± SD *	3.39 ± 1.83		
	median, [IQR *]	3 [2–4]		
Barthel index (N = 413)	<60 points	69 (16.7)		
	means ± SD	79.42 ± 21.10		
	median, [IQR]	85 [70–100]		
Pfeiffer test (N = 399)	≥3 errors [N (%)]	89 (22.3)		
	means ± SD	1.20 ± 1.98		
	median, [IQR]	0 [0–2]		
Frail index (N = 146)	≥3 points [N (%)]	26 (17.8)		
	means ± SD	1.79 ± 0.9		
	median, [IQR]	2 [1–2]		
Mini Nutritional Assessment (MNA) (N = 413)		Normonourished (12–14 points)	At risk of Malnutrition (8–11 points)	Malnourished (0–7 points)
		299 (72.4)	105 (25.4)	9 (2.2)

* IQR: interquartile range; SD: standard deviation.

**Table 2:** Characteristics of heart disease, pharmacological treatment at three months of follow-up, pre- and post-initial follow-up admissions and exitus.

Heart Failure Characteristics		N (%)
LVEF * (N = 407)	Preserved (≥50%)	308 (74.6)
	Slightly reduced (41–49%)	36 (8.7)
	Reduced (<40%)	62 (15.0)
NYHA* ≥ 3 (N = 398)		147 (36.9)
Debut heart failure (N = 413)		107 (25.9)
Echocardiography (N = 413)		401 (97.1)
Heart disease aetiology (N = 405)		N (%)
Ischaemic		116 (28.6)
Hypertensive		312 (77.0)
Valvular		181 (44.7)
Infiltrative		24 (5.9)
Valvulopathy (N = 377)		N (%)
Aortic stenosis		81 (21.5)
Aortic insufficiency		100 (26.5)
Mitral stenosis		16 (4.2)
Mitral insufficiency		215 (57.0)
Tricuspid insufficiency		153 (40.6)
Pharmacological treatment		N (%)
Beta-blockers		258 (63.5)
Angiotensin-converting enzyme inhibitors (ACE inhibitors)		40 (9.9)
Angiotensin 2 receptor antagonists (ARA2)		98 (24.1)
Angiotensin–Neprilysin Receptor Inhibitor (ARNI)		47 (11.6)
Antialdosteronics		178 (43.8)
Sodium-glucose co-transporter type 2 inhibitors (iSGLT2)		107 (26.4)
Loop diuretics		362 (89.2)
Thiazides		35 (8.6)
Anticoagulants		261 (64.3)
Anti-aggregants		109 (26.8)
Statins		220 (54.2)
Admissions and deaths		n (%)
Admission prior to follow-up for heart failure (N = 411)		258 (62.5)
Admission in year prior to start of follow-up for heart failure (N = 409)		219 (53.0)
Admission for heart failure in the first year thereafter (N = 304)		31 (7.5)
Deaths (N = 413)		256 (62.0)

* LVEF: left ventricular ejection fraction; NYHA: New York Heart Association.

**Table 3:** Differences in demographics, comorbidities, assessment scales and analytical data between normonnourished and malnourished patients or patients at risk of malnutrition.

		Total	Normonourished	Malnourished or at Risk	*p* Value	OR* (95%CI *)
Demographic Data						
Age (years)					0.445	0.97 (0.91–1.03)
Median, [IQR *]		88 [86–90]	88.0 [86–90]	88.0 [86–89]		
Means ± SD *		88.4 (±2.9)	88.4 (±3)	88.4 (±2.9)		
Sex	Males	195 (47.2%)	146 (48.8%)	49 (43%)		
	Females	218 (52.8%)	153 (51.2%)	65 (57%)	0.287	1.27 (0.82–1.96)
Lives alone		75 (19.5%)	62 (22.2%)	14 (12.7%)	0.034	0.51 (0.27–0.96)
Live in residence		8 (2.1%)	4 (1.4%)	4 (3.6%)	0.166	2.60 (0.64–10.6)
Lives with spouse		107 (29.3%)	81 (31.5%)	26 (24.1%)	0.154	0.69 (0.41–1.15)
Comorbidities						
High blood pressure		398 (96.4%)	290 (97.0%)	108 (94.7%)	0.274	0.56 (0.19–1.61)
Diabetes mellitus		195 (47.2%)	145 (48.5%)	50 (43.9%)	0.399	0.83 (0.54–1.28)
Dyslipidaemia		240 (58.1%)	181 (60.5%)	59 (51.8%)	0.106	0.70 (0.45–1.08)
Renal disease		247 (59.8%)	181 (60.5%)	66 (57.9%)	0.625	0.90 (0.58–1.39)
Stroke		50 (12.1%)	33 (11.0%)	17 (14.9%)	0.280	1.41 (0.75–2.65)
Peripheral arterial disease		30 (7.3%)	22 (7.4%)	8 (7.0%)	0.905	0.95 (0.41–2.2)
Angina		43 (10.4%)	31 (10.4%)	12 (10.5%)	0.962	1.02 (0.50–2.06)
Myocardial infarction		82 (19.9%)	53 (17.8%)	29 (25.4%)	0.082	1.58 (0.94–2.64)
Atrial fibrillation		293 (70.9%)	213 (71.2%)	80 (70.2%)	0.832	0.95 (0.59–1.52)
Hepatopathy		33 (8.0%)	22 (7.4%)	11 (9.6%)	0.443	1.34 (0.63–2.87)
COPD *		69 (16.7%)	52 (17.4%)	17 (14.9%)	0.537	0.83 (0.46–1.50)
Asthma		27 (6.5%)	23 (7.7%)	4 (3.5%)	0.124	0.44 (0.15–1.29)
Gastric ulcer		18 (4.4%)	12 (4.0%)	6 (5.3%)	0.578	1.33 (0.49–3.63)
Dementia		67 (16.2%)	31 (10.4%)	36 (31.6%)	<0.001	3.99 (2.32–6.86)
Active neoplasia		27 (6.5%)	17 (5.7%)	10 (8.8%)	0.257	1.59 (0.71–3.60)
Anaemia		240 (58.1%)	168 (56.2%)	72 (63.2%)	0.199	1.34 (0.86–2.08)
Pressure ulcer		15 (3.6%)	8 (2.7%)	7 (6.1%)	0.092	2.38 (0.84–6.72)
Hip fracture		35 (8.5%)	16 (5.4%)	19 (16.7%)	<0.001	3.54 (1.75–7.16)
Falls **≥** 3		14 (3.4%)	7 (2.3%)	7 (6.1%)	0.056	2.73 (0.93–7.96)
OSAS *		31(7.5%)	25 (8.4%)	6 (5.3%)	0.285	0.61 (0.24–1.52)
Obesity		149 (36.5%)	131 (44.6%)	18 (15.8%)	<0.001	0.23 (0.13–0.41)
Smoker		146 (36.5%)	106 (36.6%)	40 (36.4%)	0.972	0.99 (0.63–1.56)
Alcohol		82 (20.6%)	55 (19.0%)	27 (24.8%)	0.201	1.41 (0.83–2.38)
Assessment scales						
Charlson I.* (median, [IQR])		3 [2–4]	3 [2–4]	4 [2–5]	−	−
Charlson I. (means ± SD)		3.39 ± 1.83	3.23 ± 1.63	3.79 ± 2.24	<0.001	−
Charlson > 4 points, n (%)		99 (24%)	61 (20.4%)	38 (33.3%)	0.006	1.95 (1.21–3.15)
Barthel I.* (median, [IQR])		85 [70–100]	90 [75–100]	70 [50–85]	−	−
Barthel I. (means ± SD)		79.42 ± 21.10	84.08 ± 18.23	67.19 ± 23.20	0.003	−
Barthel I. <60, n (%)		69 (16.7%)	28 (9.4%)	41 (36%)	<0.001	5.44 (3.15–9.38)
Pfeiffer (median, [IQR])		0 [0–2]	0 [0–0]	2 [0–4]	−	−
Pfeiffer (means ± SD)		1.20 ± 1.98	0.74 ± 1.55	2.4 ± 2.45	<0.001	−
Pfeiffer ≥ 3 errors, n (%)		89 (22.3%)	39 (13.4%)	50 (45.9%)	<0.001	5.45 (3.29–9.04)
Frail (median, [IQR])		2 [1–2]	1 [1–2]	2 [23]	−	−
Frail (means ± SD)		1.79 ± 0.90	1.51 ± 0.73	2.32 ± 0.99	<0.001	−
Frail ≥ 3 points, n (%)		26 (17.8%)	8 (8.3%)	18 (36.0%)	<0.001	6.19 (2.45–15.61)
Analytical data (means ± SD)						
Urea (mg/dL)		79.08 (±38.88)	76.7 (±35.5)	85.29 (±46.1)	0.037	−
Creatinine (mg/dL)		1.41 (±0.61)	1.4 (±0.56)	1.4 (±0.72)	0.333	−
Glomerular filtration (mL/min)		45.30 (±18.33)	45.3 (±17.8)	45.3 (±19.8)	0.498	−
Sodium (mEq/L)		138.14 (±13.24)	137.8 (±15.4)	138.9 (±3.9)	0.236	−
Potassium (mEq/L)		4.50 (±0.60)	4.5 (±0.6)	4.5 (±0.6)	0.442	−
Total protein (g/dL)		6.70 (±0.65)	6.8 (±0.6)	6.4 (±0.7)	<0.0011	−
Albumin (g/dL)		3.73 (±0.51)	3.8 (±0.5)	3.6 (±0.5)	0.053	−
Ultrasensitive troponin T (ng/L)		55.42 (±75.64)	52.7 (±72.9)	61.6 (±81.7)	0.174	−
NT-proBNP * (pg/mL)		4991.6 (±6396.2)	4444 (±5849)	6419 (±7484)	0.008	−
Total cholesterol (mg/dL)		142.91 (±38.53)	144 (±38)	140 (±39.7)	0.179	−
TSI * (%)		18.96 (±11.75)	18.8 (±11.4)	19.3 (±12.7)	0.375	−
Ferritin (ng/mL)		149.09 (±185.55)	143.9 (±196)	162.5 (±155.3)	0.191	−

* OR: odds ratio; CI: confidence interval; IQR: interquartile range; SD: standard deviation; COPD: chronic obstructive pulmonary disease; OSAS: obstructive sleep apnoea syndrome; Charlson I.: Charlson index; Barthel I.: Barthel index; NT-proBNP: N-terminal pro-brain natriuretic peptide; TSI: transferrin saturation index.

**Table 4:** Differences in heart disease characteristics and admissions between the normonourished and the malnourished or at risk.

		Total	Normonourished	Malnourished or at Risk	*p*	OR * (95%CI *)
Cardiopathy						
LVEF *	Preserved	308 (75.7%)	229 (77.9%)	79 (69.9%)	0.093	0.66 (0.4–1.07)
	Slightly reduced	36 (8.8%)	22 (7.5%)	14 (12.4%)	0.118	1.75 (0.86–3.55)
	Reduced	62 (15.2%)	43 (14.6%)	19 (16.8%)	0.582	1.18 (0.65–2.13)
NYHA ≥ 3		147 (36.9%)	99 (34.6%)	48 (42.9%)	0.126	1.42 (0.91–2.21)
Debut HF *		107 (25.9%)	80 (26.8%)	27 (23.7%)	0.524	0.85 (0.51–1.40)
Aetiology						
Ischaemic		116 (28.6%)	80 (27.4%)	36 (31.9%)	0.373	1.24 (0.77–1.99)
Hypertensive		312 (77%)	232 (79.5%)	80 (70.8%)	0.063	0.63 (0.38–1.03)
Valvular		181 (44.7%)	116 (39.7%)	65 (57.5%)	0.001	2.05 (1.32–3.19)
Infiltrative		24 (5.9%)	20 (6.8%)	4 (3.5%)	0.206	0.50 (0.17–1.49)
Admissions						
Previous HF admissions		258 (62.8%)	182 (61.3%)	76 (66.7%)	0.312	1.26 (0.80–1.99)
Admissions due to HF in the following year		31 (10.2%)	21 (8.9%)	10 (14.7%)	0.163	1.76 (0.79–3.96)

* OR: odd ratio; CI: confidence interval; LVEF: left ventricular ejection fraction; HF: heart failure.

**Table 5:** Comparison of median survival in the normonnourished versus malnourished–risk of malnutrition group.

	Average Survival (Months)	95% Confidence Interval	*p*
Total population	31.409	(27.438–35.379)	0.001
Normonourished	35.778	(31.409–40.148)	
Malnourished or at risk	19.088	(13.460–24.717)	

## Data Availability

The data presented in this study are available on request from the corresponding author. The data are not publicly available due to privacy restrictions.
